# Lysophosphatidic acid promotes cutaneous wound healing through vascular maturation and fibroblast activation in a murine model

**DOI:** 10.1186/s41232-026-00441-5

**Published:** 2026-08-28

**Authors:** Kazuhiro Takara, Aika Yamamoto, Naoi Hosoe, Miku Aoki, Takenao Chino, Takayuki Sonoda, Shintaro Yamada, Yumiko Hayashi, Lamri Lynda, Minoru Hasegawa, Junko Yotsuya, Hiroyasu Kidoya

**Affiliations:** 1 Department of Integrative Vascular Biology, Faculty of Medical Sciences, University of Fukui, 23-3 Matsuoka-Shimoaizuki, Eiheiji, Yoshida, Fukui, 910-1193 Japan; 2 Tenure-Track Program for Innovative Research, University of Fukui, Fukui, Japan; 3 Division of Nursing, Faculty of Medical Sciences, University of Fukui, Fukui, Japan; 4 Department of Gerontological Nursing/Wound Care Management, Graduate School of Medicine, The University of Tokyo, Tokyo, Japan; 5 Department of Dermatology, Faculty of Medical Sciences, University of Fukui, Fukui, Japan; 6 Department of Endocrinology and Metabolism, School of Medical Sciences, University of Fukui, Fukui, Japan; 7 Department of Neurosurgery, Division of Medicine, Faculty of Medical Sciences, University of Fukui, Fukui, Japan

**Keywords:** Lysophosphatidic acid, Wound healing, Angiogenesis, Vascular maturation, Fibroblast, Lymphangiogenesis, LPA receptor

## Abstract

**Background:**

Impaired cutaneous wound healing is a major clinical burden, particularly among older individuals and patients with diabetes. Lysophosphatidic acid (LPA) is a bioactive phospholipid with known roles in cell proliferation, migration, and vascular formation; however, its mechanistic contribution in wound repair has not been systematically characterized. Therefore, this study aimed to determine whether topical LPA accelerates wound closure in a murine full-thickness excisional wound model and to elucidate the underlying cellular and molecular mechanisms.

**Methods:**

Full-thickness excisional wounds were created on the dorsal skin of mice, and LPA or vehicle was applied once daily. Wound closure was assessed over 13 days using digital planimetry. Bulk RNA sequencing, quantitative reverse transcription polymerase chain reaction, immunofluorescence, flow cytometry, vascular permeability, and tissue hypoxia assays were employed to delineate the underlying mechanisms. Published single-cell RNA sequencing data from mouse skin were re-analyzed to map LPA receptor expression.

**Results:**

Topical LPA accelerated the closure of full-thickness excisional skin wounds. Transcriptomic analysis identified upregulation of gene programs associated with vasculature development and extracellular matrix organization. LPA markedly increased endothelial cell numbers and vessel density. Critically, the vessels formed in LPA-treated wounds subsequently displayed elevated pericyte coverage, reduced permeability, and decreased tissue hypoxia, indicating functional vascular maturation. Concurrently, fibroblast-associated matrix genes were upregulated, collagen deposition was accelerated, and lymphangiogenesis was promoted at the wound site. Single-cell analysis implicated LPAR1 in fibroblasts and LPAR4/LPAR6 in endothelial cells as candidate mediators of these effects.

**Conclusions:**

LPA promotes both vascular maturation and fibroblast activation during wound repair, distinguishing it from classical pro-angiogenic factors. These findings establish LPA as a mechanistically distinct therapeutic candidate and suggest a novel approach for targeting vascular maturation in cutaneous wound care.

**Supplementary Information:**

The online version contains supplementary material available at https://doi.org/10.1186/s41232-026-00441-5.

## Background

Cutaneous wound healing is a highly orchestrated process that encompasses hemostasis, inflammation, proliferation, and tissue remodeling. Disruption of any phase can result in chronic, non-healing wounds, a problem that is disproportionately prevalent among older individuals and patients with diabetes mellitus or peripheral vascular disease [1, 2]. The global burden of chronic wounds is substantial, contributing to prolonged hospitalization, limb loss, and markedly impaired quality of life. Angiogenesis plays a central role in tissue repair during the proliferative phase. Newly formed blood vessels supply oxygen and nutrients to the expanding granulation tissue, thereby sustaining the proliferation of fibroblasts and keratinocytes, which are essential for matrix deposition and re-epithelialization [1, 3]. Without adequate vascular ingrowth, the wound microenvironment becomes hypoxic, impairing cellular metabolism and stalling the healing process. The quality of the neovasculature is therefore as critical as its quantity.

Several growth factor-based therapies, such as fibroblast growth factor (FGF), platelet-derived growth factor (PDGF), and epidermal growth factor (EGF), targeting the proliferative phase have been clinically evaluated. These agents primarily accelerate granulation tissue formation and re-epithelialization by activating fibroblasts and keratinocytes [4]. Vascular endothelial growth factor (VEGF) is the most potent inducer of angiogenesis that robustly stimulates endothelial cell (EC) proliferation; however, the resulting vessels are often structurally immature and highly permeable, potentially impairing, rather than supporting tissue perfusion [5]. Functional vasculature requires EC expansion as well as pericyte recruitment, which stabilizes vessel walls, reduces permeability, and ensures efficient blood flow. This process, termed vascular maturation, has been insufficiently targeted by existing therapeutic strategies, representing an unmet clinical need.

Lysophosphatidic acid (LPA) is a bioactive phospholipid that signals through six cognate G protein-coupled receptors (LPAR1-6) expressed across diverse cell types [6–8]. LPA exerts pleiotropic effects such as stimulation of fibroblast proliferation, epithelial cell migration, and EC survival [6, 9]. Several studies have reported that LPA promotes wound-relevant cellular behaviors in vitro, including keratinocyte migration and fibroblast activation [10, 11]. However, these investigations were largely confined to isolated cell systems, and a comprehensive mechanistic characterization of LPA action in an established in vivo wound model is lacking. Therefore, it remains unclear whether LPA can coordinate the multiple cellular processes required for effective tissue repair, and whether it can promote the formation of functionally mature vasculature in the wound context.

Our group has previously demonstrated that LPA promotes vascular maturation in the context of tumor biology. In a subcutaneous tumor model, we showed that LPA signaling through LPAR4 promotes the formation of fine vascular networks by inducing circumferential actin bundling and linear adherens junction formation via VE-cadherin in ECs, thereby reducing vascular permeability and improving drug delivery [12]. Subsequent work from our group extended these findings to brain tumors, where LPAR4-mediated vascular network formation facilitated RhoA/ROCK-dependent tightening of endothelial cell–cell contacts, enhanced lymphocyte infiltration, and augmented the efficacy of anti-PD-1 immunotherapy [13]. Collectively, these studies established that LPA, acting primarily through LPAR4, normalizes structurally and functionally aberrant tumor vasculature, rather than simply expanding it. This concept of LPA-driven vascular normalization prompted us to investigate whether analogous vessel-stabilizing effects could be harnessed in the wound microenvironment, where immature and leaky neovessels are recognized as obstacles to efficient tissue repair.

Therefore, in the present study, we employed a murine full-thickness excisional wound model to determine whether topical LPA accelerates wound closure and to elucidate the underlying cellular and molecular mechanisms. We report that LPA simultaneously induces vascular maturation and fibroblast activation, thereby establishing a conceptually distinct mechanism of action from that of existing therapies.

## Methods

### Animals and wound model

Eight-week-old female C57BL/6 mice were obtained from Sankyo Labo Service Corporation (Tokyo, Japan) and used throughout the study. Three days before wounding, dorsal hair was removed using a depilatory cream. On day 0, a single full-thickness excisional wound (8-mm biopsy punch) was created under isoflurane anesthesia [14]. LPA (oleoyl-LPA; 18:1; Avanti Polar Lipids, Alabaster, AL, USA) was dissolved in vehicle (phosphate-buffered saline) at 0.1, 0.3, or 1.0 mg/mL. The solution (approximately 30 μL) was applied topically as drops, absorbed for 30 min, gently blotted, and covered with a Tegaderm dressing; dosing was repeated daily. In a separate delayed-treatment cohort, topical LPA (1.0 mg/mL) or vehicle was applied once daily beginning on day 3 after wounding. To evaluate the contribution of endogenous LPA production to wound healing, a separate cohort of wounded mice was treated orally with the autotaxin (ATX) inhibitor PF-8380 (30 mg/kg; #S8218, Selleck Chemicals, Houston, TX, USA) or vehicle (0.5% carboxymethyl cellulose sodium salt [CMC-Na]) once daily beginning on day 0. Wound images were acquired under standardized illumination, and the wound area was measured using ImageJ and expressed as percentage of day-0 area. All procedures were approved by the Institutional Animal Care and Use Committee of the University of Fukui (approval number: R08019), in accordance with the Guide for the Care and Use of Laboratory Animals. All animal experiments were conducted in compliance with the ARRIVE guidelines, as well as institutional and national guidelines for the care and use of laboratory animals.

### Histology and immunofluorescence

Histological analyses were performed within days 5–9, corresponding to the period during which the difference in wound closure between the LPA and vehicle groups was most pronounced. CD31 staining was performed on day 7, when vessel formation was sufficient for reliable quantification. H&E and Masson's trichrome staining were performed on day 9, when morphological differences between the groups were most pronounced. FITC-dextran permeability and hypoxia assays were performed on day 13 because phagocytic uptake of dextran at day 7 confounded quantification. Skin at the wound site was harvested on the indicated days and fixed in 4% paraformaldehyde. Cryosections (30 μm) were used for immunofluorescence. Cryosections (5 μm) were also used for hematoxylin and eosin (H&E) and Masson’s trichrome staining. For immunofluorescence, sections were blocked in 5% normal donkey serum and incubated with primary antibodies: rat anti-CD31 (#550,274, clone MEC13.3; BD Biosciences, San Jose, CA, USA), Armenian hamster anti-CD31 (#MAB1398Z, clone 2H8; Merck Millipore, Burlington, MA, USA), mouse anti-αSMA-Cy3 (#C6198, clone 1A4; Sigma-Aldrich, St. Louis, MO, USA), rabbit-anti K14 (#905,303, clone Poly19053; Biolegend, San Diego, CA, USA), rabbit anti-ERG (#ab92513, clone EPR3864; Abcam, Cambridge, UK), rat anti-Ki67 (#14–5698-82, clone SolA15; Invitrogen/eBioscience, Thermo Fisher Scientific, Waltham, MA, USA), rabbit polyclonal anti-NG2 (#AB5320; Merck Millipore), rat anti-ER-TR7 (#NBP1-44,961; Novus Biologicals, Centennial, CO, USA), and rat anti-VE-cadherin (#138,001, clone BV13; Biolegend). Species-matched Alexa Fluor-conjugated secondary antibodies (Thermo Fisher Scientific) were applied [15]. Rat anti-CD31 was used for single immunostaining and for co-immunostaining with rabbit-derived primary antibodies, whereas hamster anti-CD31 was used for co-immunostaining with other rat-derived primary antibodies (e.g., anti-ER-TR7) to avoid species cross-reactivity. Images were acquired using a confocal laser scanning microscope (Leica STELLARIS 5, Leica Microsystems, Wetzlar, Germany) and a widefield/confocal Microhub (Leica Mica), and analyzed using Leica LAS X software, Fiji/ImageJ and AngioTool [16]. Vessel density was quantified as percentage of CD31-positive area within the granulation tissue region. ERG (CD31) and NG2 (CD31) structures were enumerated manually in randomly selected fields by a blinded investigator.

### Flow cytometry

Skin at the wound site (diameter, 8 mm) was minced and enzymatically dissociated into a single-cell suspension. Tissue fragments were digested with several enzymes, including dispase (#17,105- 041, Gibco, Waltham, MA) and collagenases (#034–22363, Wako, Osaka, Japan, and #LS004176, Worthington Industries, Columbus, OH, USA), followed by mechanical dissociation using a gentleMACS Octo Dissociator (Miltenyi, Bergisch Gladbach, Germany) to prepare single-cell suspensions. The resulting suspension was filtered through 70 µm strainers, pelleted (300 × g, 10 min, 4 °C), and resuspended in phosphate buffered saline containing 2% fetal bovine serum. Single-cell suspensions were stained with Zombie NIR Viability Dye (BioLegend), anti-CD31-BV421 (clone 390; BD Biosciences), and anti-CD45-PE (clone 30-F11; BioLegend). ECs were identified as CD31⁺CD45⁻ events within the live single-cell gate. Data were acquired using a Sony SH800S cell sorter (SONY, Tokyo, Japan) and analyzed using FlowJo v10 (BD Biosciences).

### Vascular permeability and tissue hypoxia assays

For permeability assessment, 70 kDa FITC-dextran (Sigma-Aldrich; 25 mg/kg) was injected intravenously 30 min before tissue harvesting on day 13. Cryosections were co-stained with an anti-CD31 antibody, and the ratio of FITC-dextran fluorescence to the CD31 signal was quantified as an index of extravasation. Tissue hypoxia was assessed via intraperitoneal injection of 60 mg/kg pimonidazole hydrochloride (Hypoxyprobe-1; Burlington, MA, USA) 2 h before harvest [17]. Pimonidazole adducts were detected using an anti-pimonidazole antibody (Hypoxyprobe Inc.) and quantified relative to the CD31 signal.

### RNA sequencing and quantitative reverse transcription polymerase chain reaction (qRT-PCR)

Total RNA was extracted from the skin at the wound site using QIAzol Lysis Reagent (QIAGEN, Hilden, Germany) and purified using an RNeasy Mini Kit (Qiagen). For bulk RNA sequencing, libraries were prepared using the TruSeq Stranded Total RNA Library Prep kit and sequenced on an Illumina NovaSeq 6000 (150 bp paired-end reads; n = 3 per group per time point). The RNA-sequencing (RNA-seq) data were analyzed using iDEP (ver2.5.2). Differentially expressed genes (DEGs) were identified using DESeq2 with a maximum false discovery rate-adjusted p < 0.05 and a 1.5-fold change between groups [18–20]. The RNA-seq data are available in the Gene Expression Omnibus (accession code: PRJDB42128). For quantitative RT-PCR, cDNA was synthesized using the PrimeScript RT Reagent Kit (Takara Bio, Shiga, Japan), and the expression of fibronectin, Col1a1, Col3a1, and Tgfb was quantified using TB Green Premix Ex Taq (Takara Bio) and normalized to Gapdh. The following primer sequences were used: fibronectin forward [CTACCCTGCAGCCTCTGCGC] and reverse [TCACCTCCCTGGCTCGGTCG]; Col1a1 forward [TGTTCGTGGTTCTCAGGGTAG] and reverse [TTGTCGTAGCAGGGTTCTTTC]; Col3a1 forward [TGCCCACAGCCTTCTACACCT] and reverse [CCAGCTGGGCCTTTGATACCT]; Tgfb forward [GGATACCAACTATTGCTTCAGCTCC] and reverse [AGGCTCCAAATATAGGGGCAGGGTC]; Enpp2 forward [GGATTACAGCCACCAAGC AAGG] and reverse [ATCAGGCTGCTCGGAGTAGAAG]; Lpar1 forward [GGACACCAT GATGAGCCTTCTG] and reverse [TCTCATAGGCCAGGACATCGCA]; Gapdh forward [TGTGTCCGTCGTGGATCTGA] and reverse [TTGCTGTTGAAGTCGCAGGAG].

### Single-cell RNA sequencing re-analysis

Publicly available single-cell RNA sequencing data from whole mouse skin, comprising both unwounded and wounded conditions, were obtained from the Gene Expression Omnibus (GEO) database under accession number GSE142471 and re-analyzed using Seurat v5.0.1 in R v4.3.2. Data from individual samples were integrated using the Harmony algorithm implemented in the Harmony R package v1.2.0 to correct for batch effects while preserving biological variation. Dimensionality reduction and visualization were performed using Uniform Manifold Approximation and Projection (UMAP). Cell clusters were annotated based on the expression of canonical marker genes, together with the cell-type annotations provided in the original study by Haensel et al. [21]. Lpar1–6 transcript expression was visualized using feature plots overlaid on the UMAP embedding. The endothelial cell cluster was computationally isolated and re-clustered to resolve EC subclusters, which were annotated using established marker genes for capillary (*Rgcc*), arterial (*Gja5*), venous (*Ackr1*), proliferating (*Mki67*), and tip-like (*Esm1*) ECs.

### Statistical analysis

Data are presented as mean ± SD. Two-group comparisons were performed using two-tailed unpaired Student’s *t*-tests. Multi-group, multi-time-point analyses were performed using two-way ANOVA with Tukey’s multiple comparison correction. All analyses were conducted in GraphPad Prism 10, and p < 0.05 was considered statistically significant.

## Results

### Topical LPA accelerates full-thickness wound closure

**Fig. 1:**
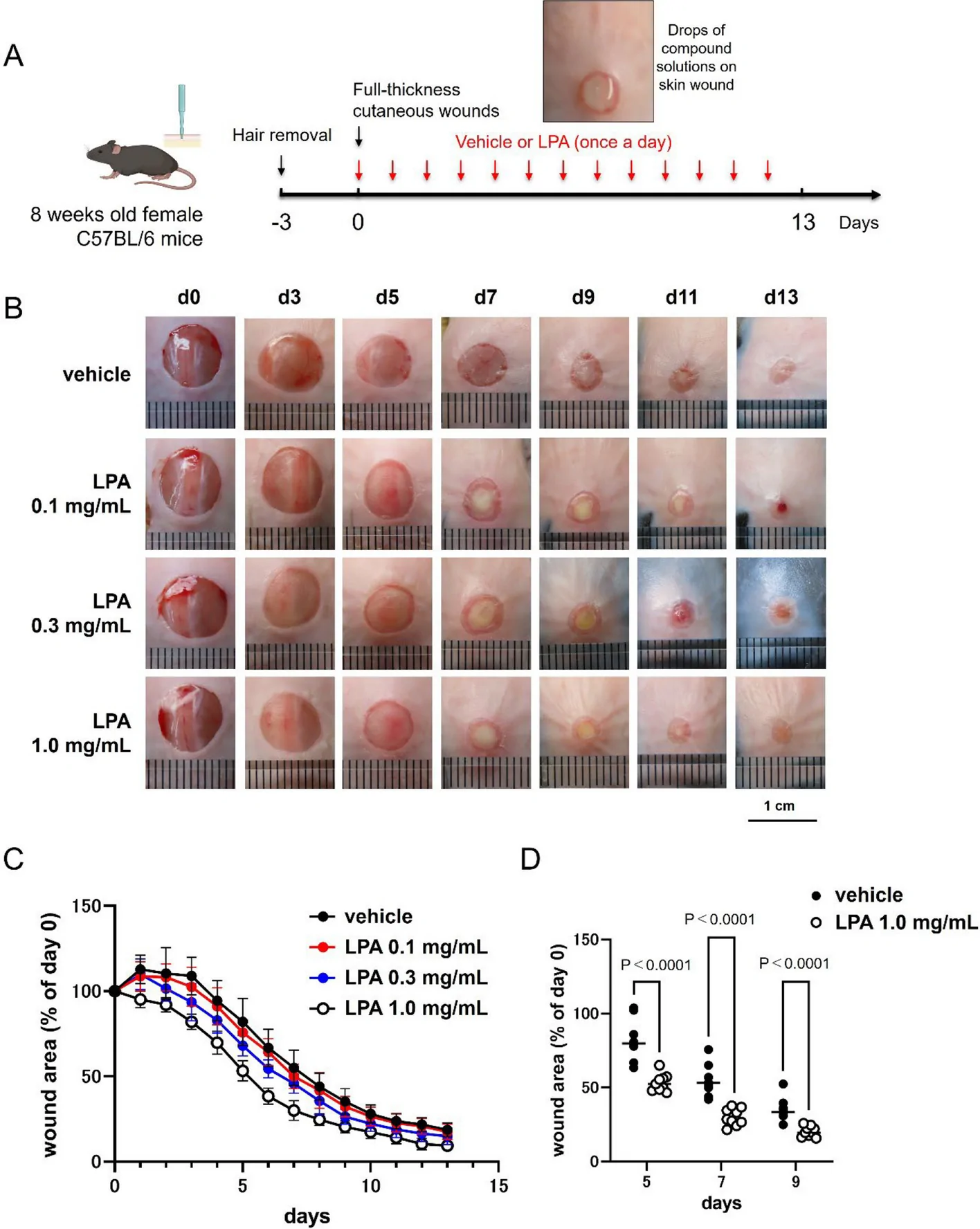
Topical LPA accelerates cutaneous wound closure. **A** Experimental timeline. Female C57BL/6 mice (8 weeks old) underwent dorsal hair removal on day − 3. Full-thickness excisional wounds (8-mm punch biopsy) were created on day 0. LPA (0.1, 0.3, or 1.0 mg/mL) or vehicle was applied topically once daily. Right panel: representative photograph showing topical LPA administration. **B** Representative wound photographs of the same mouse in each treatment group, acquired on days 0, 3, 5, 7, 9, 11, and 13. Scale bar = 1 cm. **C** Time-course of wound area normalized to day 0 area (mean ± SD; n = 7–9 per group). **D** Scatter plots of individual wound areas at days 5, 7, and 9 for the vehicle and LPA 1.0 mg/mL groups; P < 0.0001

To investigate the therapeutic potential of LPA in cutaneous wound repair, mice were delivered a full-thickness excisional wound on day 0, followed by daily topical application of LPA (0.1, 0.3, or 1.0 mg/mL) or vehicle (Fig. 1A, B). The wounded area was monitored for 13 days. All three LPA concentrations showed an accelerating trend relative to the vehicle, and the 1.0 mg/mL group exhibited the most pronounced reduction in wound area (Fig. 1C). Wound area differed significantly between the LPA-treated and vehicle-treated groups throughout the observation period. Among the three doses tested, the greatest acceleration of wound closure was observed with the highest LPA concentration (1.0 mg/mL). To focus on the proliferative phase, where the difference in wound size between the groups was the most pronounced, we selected days 5, 7, and 9 for quantitative comparison; all three time points showed marked differences between the LPA 1.0 mg/mL group and vehicle group (Fig. 1D). Because clinical wounds are often not treated immediately after injury, we next investigated whether LPA remains effective when treatment is initiated after a delay. When topical LPA (1.0 mg/mL) or vehicle was applied once daily beginning on day 3 after wounding, LPA still significantly accelerated wound closure relative to the vehicle at days 5, 7, and 9 (Supplementary Fig. 4), indicating that its pro-healing effect is maintained even when treatment is initiated several days after injury.

We next investigated whether wounding itself induces endogenous LPA production. Because LPA cannot be measured directly at the transcript level, we used expression of its biosynthetic enzyme, autotaxin (ATX, encoded by *Enpp2*), as a surrogate marker. Re-analysis of a published single-cell RNA-seq dataset showed *Enpp2* expression in endothelial cells, fibroblasts, myofibroblasts, and epidermal basal cells (Supplementary Fig. 1A). Consistently, qRT-PCR revealed a sharp early peak in *Enpp2* expression at 3–6 h post-wounding, followed by a return to near-baseline levels by day 1 (Supplementary Fig. 1B), indicating a rapid and transient induction of ATX expression after wounding. To evaluate whether endogenous LPA induced by wounding contributes to wound healing, we orally administered the ATX inhibitor PF-8380. This resulted in a modest but statistically significant delay in wound closure on day 1 (*P* = 0.041), with a non-significant trend toward delayed healing at later time points (Supplementary Fig. 1C–E). These findings suggest that endogenous ATX-derived LPA contributes to the early phase of wound healing.

### LPA upregulates gene programs associated with vascular development and extracellular matrix (ECM) organization

**Fig. 2:**
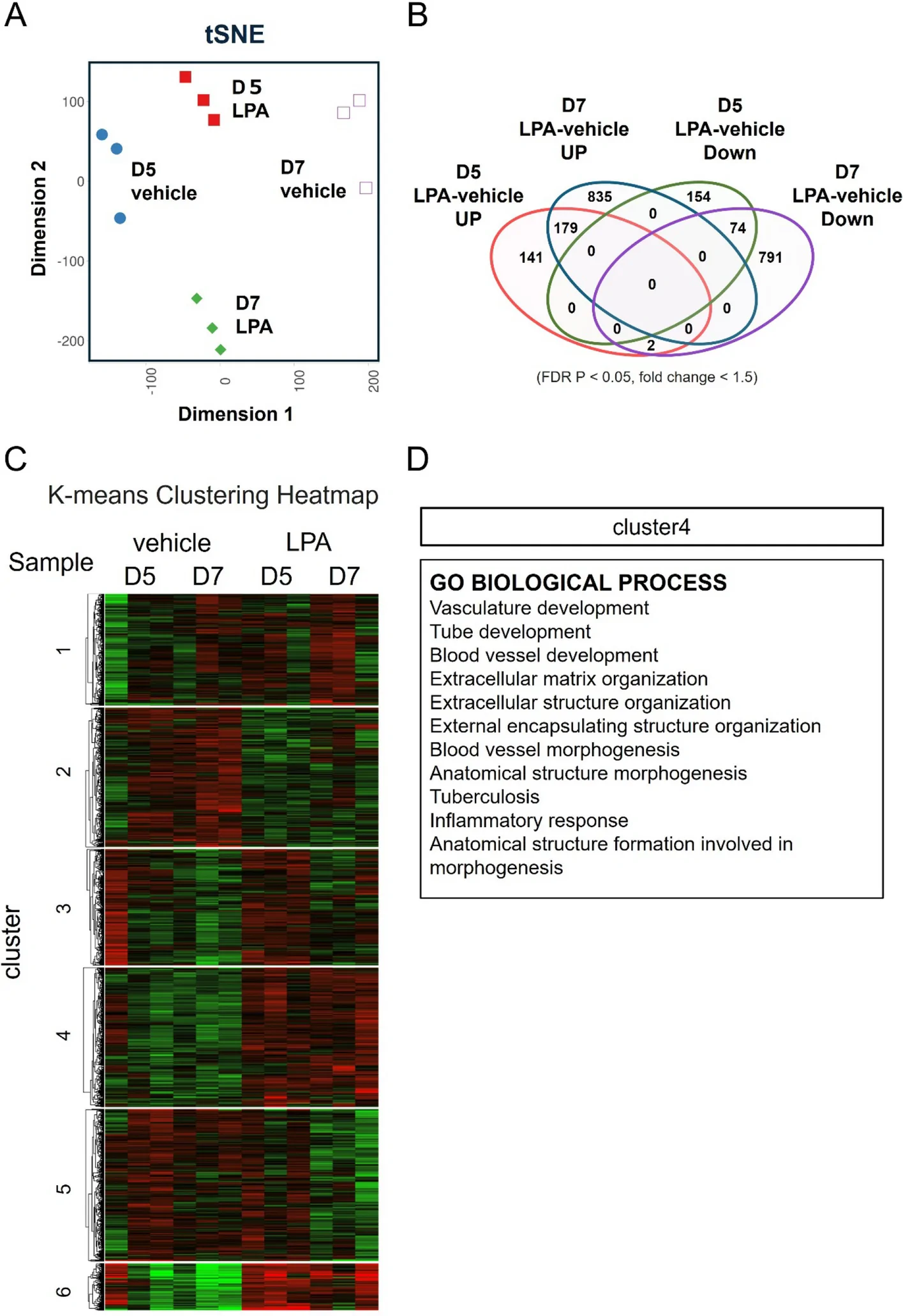
LPA upregulates angiogenic and ECM gene programs at the wound site. **A** tSNE plot of bulk RNA sequencing samples from the skin at wound site harvested on days 5 and 7 from vehicle- and LPA 1.0 mg/mL-treated mice (n = 3 per group per time point). Each symbol represents one biological replicate. **B** Venn diagram of differentially expressed genes (false discovery rate < 0.05, fold change ≥ 1.5) upregulated (UP) or downregulated (Down) by LPA vs. vehicle at day 5 and day 7. **C** K-means clustering heatmap (k = 6) of all differentially expressed genes. Color scale represents row-normalized expression (z-score). **D** Representative GO (Gene Ontology) Biological Process enrichment terms for cluster 4, which contains genes upregulated by LPA at both day 5 and day 7

To characterize the transcriptional basis of LPA-mediated healing, total RNA from the skin at wound site on days 5 and 7 was subjected to bulk RNA sequencing. *T*-distributed stochastic neighbor embedding (tSNE) visualization revealed distinct clustering of samples at both the treatment and time points (Fig. 2A). Differential expression analysis revealed that 322 genes were upregulated on day 5, and 1,014 genes were upregulated on day 7, with 179 genes shared between both time points. Downregulated genes included 154 genes that were unique to day 5 and 791 unique to day 7, with 74 genes shared between both time points (Fig. 2B). K-means clustering identified six distinct gene modules (Fig. 2C). Cluster 4, which was consistently elevated in LPA-treated samples at both time points, was enriched in GO Biological Process terms, including vasculature development, tube development, blood vessel morphogenesis, ECM organization, and inflammatory response (Fig. 2D). These data indicate that LPA coordinately activates angiogenic and matrix remodeling transcriptional programs during the proliferative phase.

### LPA promotes angiogenesis and induces vascular maturation at the wound site

**Fig. 3:**
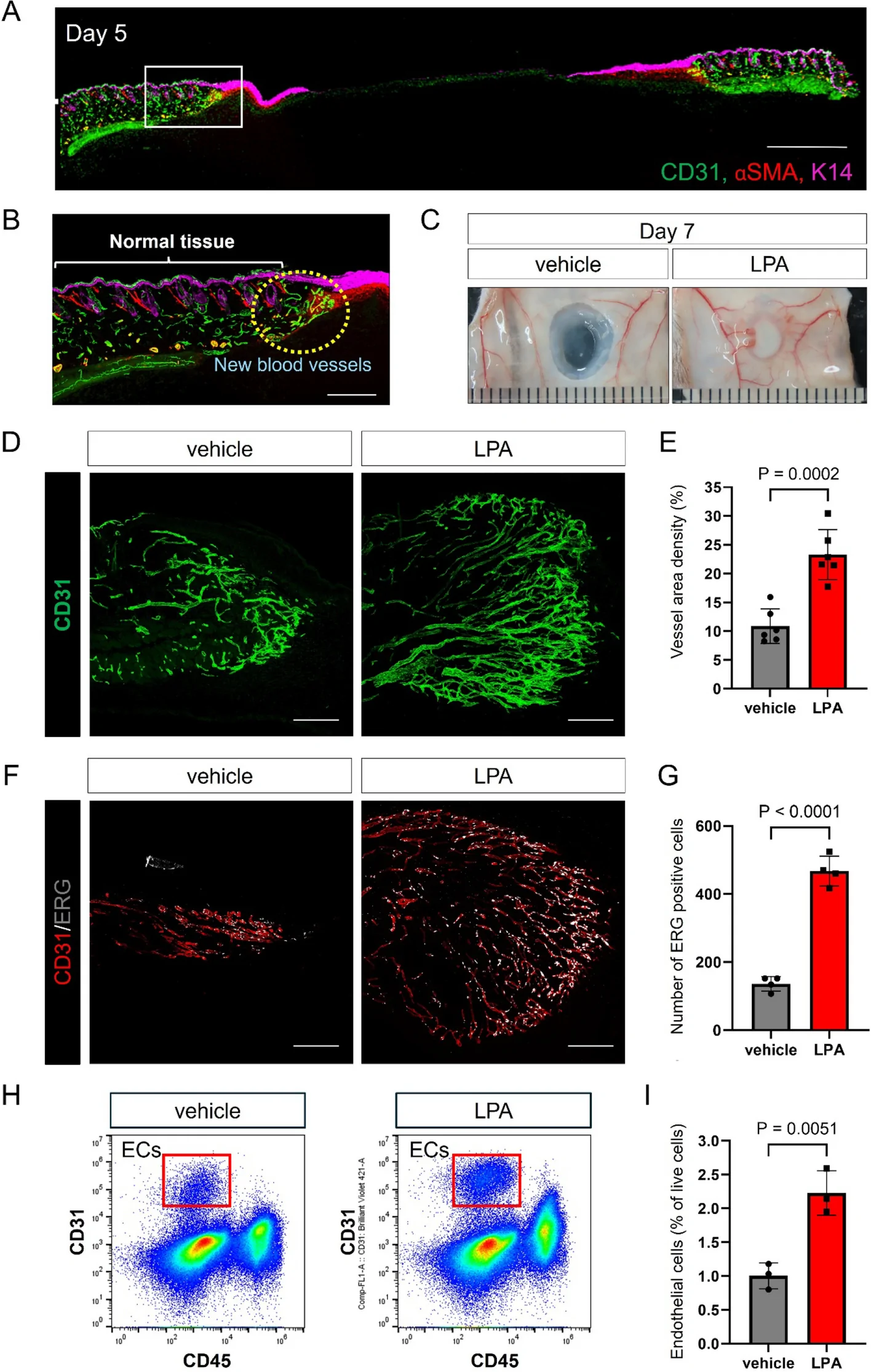
LPA increases endothelial cell number and vessel density at the wound site. **A** Representative low-magnification immunofluorescence image of a day 5 wound section stained for CD31 (green), αSMA (red), and K14 (magenta). Scale bar = 1 mm. **B** Enlarged view of the boxed region in (**A**) highlighting newly formed vasculature within granulation tissue. Scale bar = 300 μm. **C** Macroscopic photographs of day 7 wounds. **D** Representative confocal images of CD31-stained day 7 wound sections from vehicle- and LPA-treated mice. Scale bar = 200 μm. **E** Vessel density (percentage of CD31-positive area within the granulation tissue region) quantified from (**D**). n = 6; P = 0.0002. **F** Representative confocal images of CD31/ERG co-stained day-7 wound sections. Scale bar = 200 μm. **G** Number of ERG⁺CD31⁺ endothelial cells per field, quantified from (**F**). n = 4; P < 0.0001. **H** Representative flow cytometry dot plots showing endothelial cell (EC; CD31⁺CD45⁻) gating following sequential scatter (All), singlet (Single), and viability (Live) gates. **I** Percentage of ECs among live cells quantified from flow cytometry analysis. n = 3; P = 0.0051. Data are presented as mean ± SD

Histological examination of day 5 wound sections immunostained for CD31 (ECs), αSMA (smooth muscle cells/myofibroblasts), and K14 (keratinocytes) provided an overview of the newly forming granulation tissue (Fig. 3A). Higher-magnification views of the wound edge revealed accumulation of newly formed blood vessels at the advancing front of regenerated tissue (Fig. 3B). Macroscopic photographs of day 7 wounds revealed a far denser and more regularly oriented surface vasculature in LPA-treated mice than in vehicle-treated mice (Fig. 3C). Confocal imaging of CD31-stained sections confirmed a higher vessel density on day 7 in LPA-treated wounds (Fig. 3D), quantified as a significant increase in the percentage of CD31-positive area (Fig. 3E). Co-staining for the endothelial-specific transcription factor ERG and CD31 demonstrated a markedly higher number of ERG⁺CD31⁺ cells per field (Fig. 3F, G). Flow cytometric analysis of dissociated wound tissue confirmed a significant increase in the proportion of CD31⁺CD45⁻ ECs among live single cells (Fig. 3H, I).

**Fig. 4:**
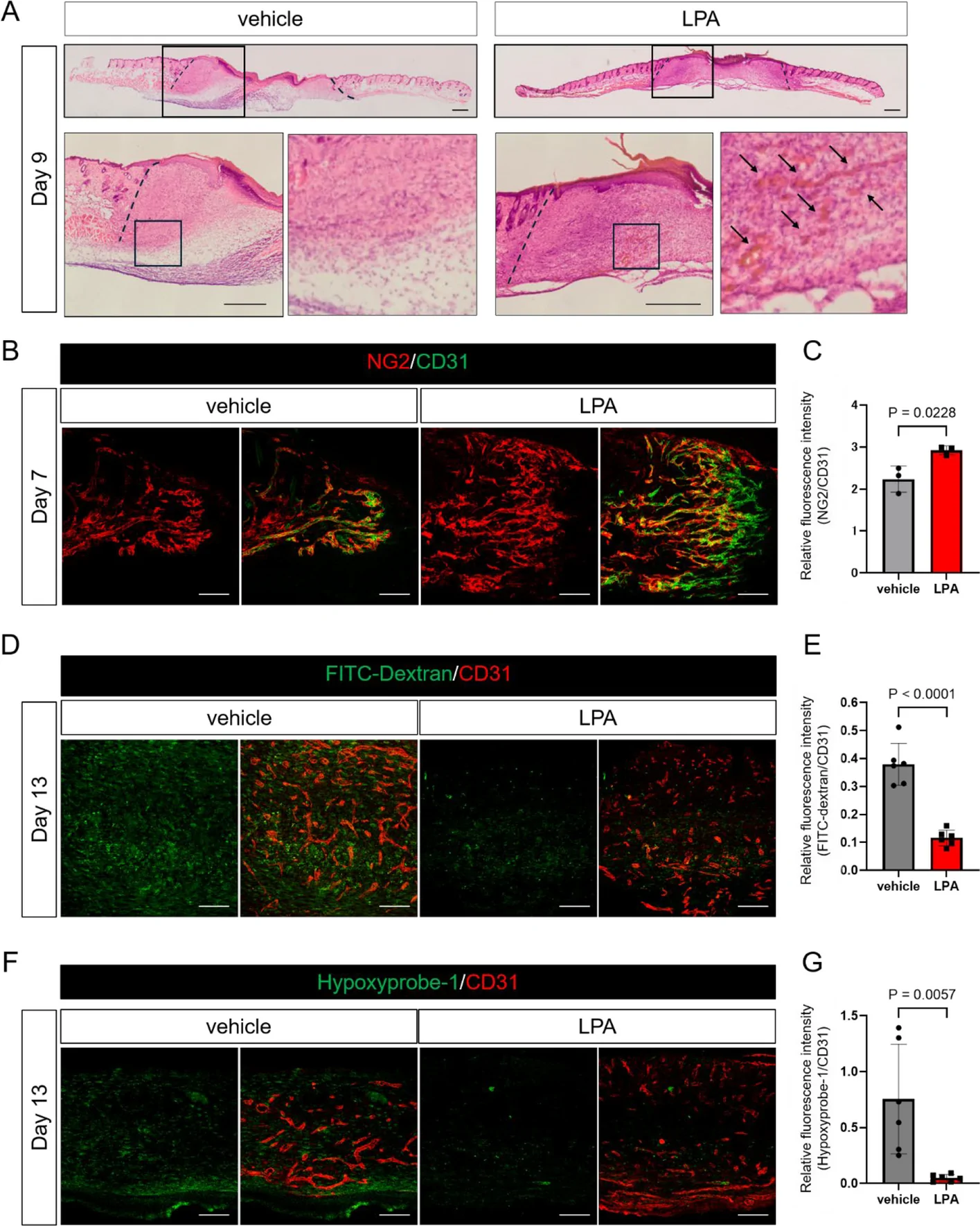
LPA induces vascular maturation and reduces tissue hypoxia. **A** Representative H&E images of day 9 wound sections. Dashed lines delineate the boundary between the wound area and the adjacent normal skin. Lower panels show higher-magnification views of boxed regions. Arrows indicate morphologically mature blood vessels in LPA-treated tissue. Scale bar = 500 μm. **B** Representative confocal images of NG2 (red)/CD31 (green) co-stained day 7 wound sections. Scale bar = 100 μm. **C** Number of NG2⁺ (pericyte-positive) CD31⁺ vessels per field, quantified from (**B**); n = 3; P = 0.0228. **D** Representative confocal images of intravenously administered FITC-dextran (green) extravasated from blood vessels into the surrounding tissue in day-13 wound sections, co-stained with CD31 (red). Scale bar = 100 μm. **E** Quantification of vascular permeability, expressed as the ratio of extravascular FITC-dextran fluorescence intensity to CD31 signal intensity per field; n = 6; P < 0.0001. **F** Representative confocal images of Hypoxyprobe-1 (green)/CD31 (red) co-stained day 13 wound sections. Scale bar = 100 μm. **G** Quantification of tissue hypoxia, expressed as the ratio of Hypoxyprobe-1 fluorescence intensity to CD31 signal intensity per field, reflecting the extent of hypoxic area relative to vascular density; n = 6; P = 0.0057. Data are presented as mean ± SD

To determine whether the expanded vasculature was also functionally mature, we assessed pericyte coverage, vascular permeability, and tissue oxygenation. H&E staining of day 9 wound sections revealed thicker-walled, morphologically mature blood vessels in LPA-treated tissue (Fig. 4A, arrows), which were largely absent in the vehicle tissue. Co-immunostaining for NG2, a marker of pericytes, and CD31 showed elevated pericyte coverage, as assessed through the NG2/CD31 fluorescence intensity ratio on day 7 (Fig. 4B, C). Line-scan fluorescence intensity profiling across vessel walls further showed that CD31 and VE-cadherin signals were poorly aligned in vehicle-treated wounds but closely coincided along the endothelial membrane in LPA-treated wounds (Supplementary Fig. 2A, B), indicating that LPA promotes the assembly of stabilized VE-cadherin–based adherens junctions. Vascular permeability was evaluated by measuring the extravasation of intravenously administered FITC-dextran from blood vessels into the surrounding tissue, and leakage was significantly lower in LPA-treated wounds on day 13 (Fig. 4D, E). Tissue hypoxia, identified via hypoxyprobe staining, which labels hypoxic cells in situ, was markedly reduced in the LPA-treated wounds on day 13 (Fig. 4F, G). Together, these results demonstrate that LPA expands the EC compartment and also drives the formation of structurally and functionally mature vasculature associated with improved wound oxygenation.

### LPA promotes granulation tissue formation and lymphangiogenesis

**Fig. 5:**
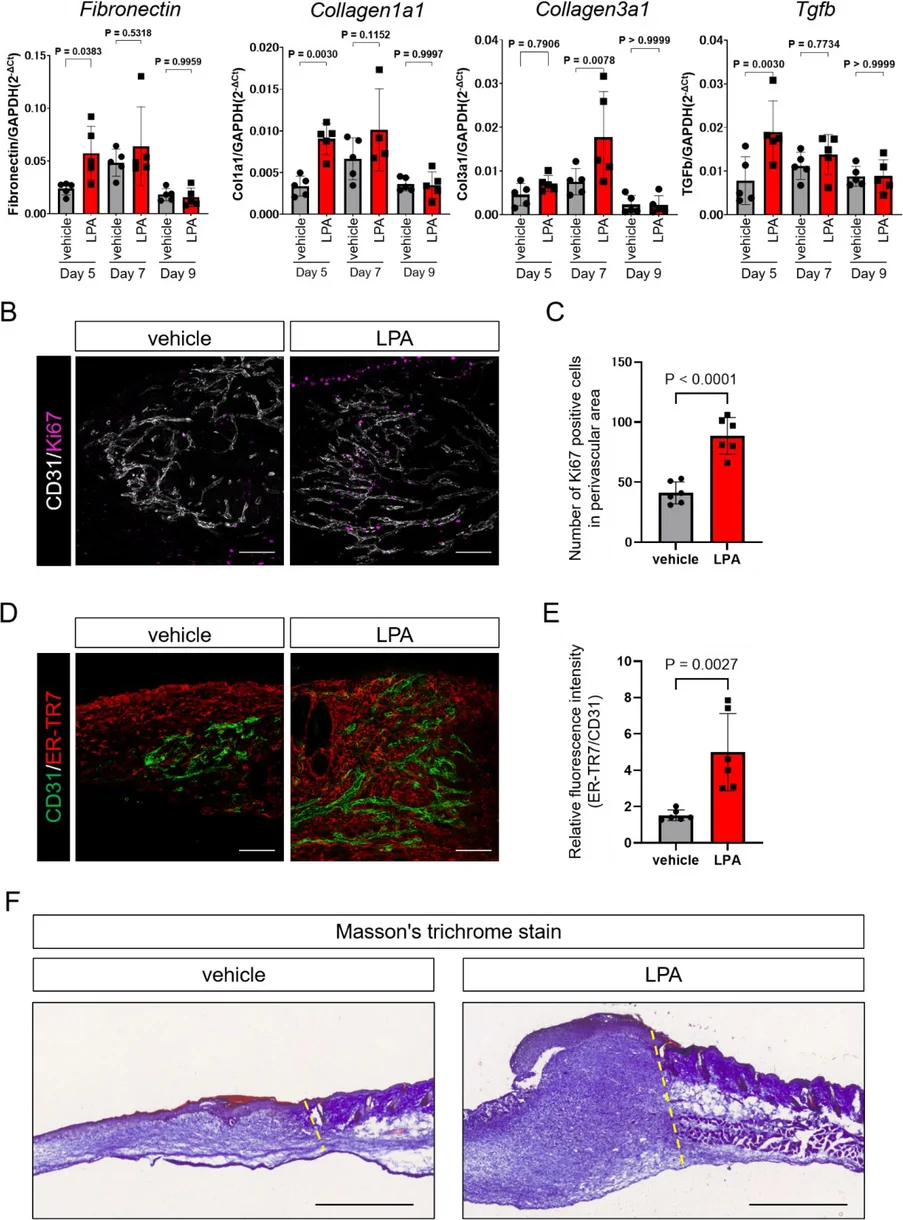
LPA activates fibroblasts and promotes granulation tissue formation. **A** Relative mRNA expression of fibronectin, Col1a1, Col3a1, and Tgfb (normalized to Gapdh) in wound tissue at days 5, 7, and 9; n = 4–5 per group. **B** Representative confocal images of CD31/Ki67 co-stained day 9 wound sections. Scale bar = 100 μm. **C** Quantification of Ki67-positive proliferating cells in the perivascular space per field, quantified from (**B**); n = 6; P < 0.0001. **D** Representative confocal images of CD31/ER-TR7 co-stained day 9 wound sections. Scale bar = 100 μm. **E** Quantification of ER-TR7⁺ fibroblast density relative to CD31 signal per field, quantified from (**D**); n = 6; P = 0.0027. **F** Masson's trichrome-stained whole-wound sections at day 9, showing collagen fiber deposition (blue) in vehicle- and LPA-treated wounds. Dashed lines delineate the boundary between the wound area and the adjacent normal skin. Scale bar = 500 μm. Data are presented as mean ± SD

Given the transcriptomic enrichment of ECM organization in cluster 4 (Fig. 2D), we examined fibroblast gene expression and granulation tissue deposition. Quantitative RT-PCR of wound tissue on days 5, 7, and 9 revealed significant upregulation of fibronectin, Col1a1, and Tgfb on day 5 and of Col3a1 on day 7, all returning to baseline by day 9 (Fig. 5A). To determine whether this transient response reflected a corresponding decline in fibroblast responsiveness to LPA, we quantified Lpar1 expression at the same time points. Lpar1 expression did not decrease significantly between day 5 and day 9 (Supplementary Fig. 3), indicating that the decline in matrix gene response is unlikely to result from the loss of Lpar1 expression. This early peaking pattern suggests a transient, but productive fibroblast activation response. Co-immunostaining for Ki67 and CD31 revealed an increased number of proliferating cells in the perivascular region of LPA-treated wounds (Fig. 5B, C). Co-staining with the fibroblast markers ER-TR7 and CD31 confirmed the increased abundance of fibroblasts in the perivascular region (Fig. 5D, E). Taken together, these findings are consistent with LPA promoting fibroblast expansion in proximity to the maturing vasculature, suggesting that the improved tissue oxygenation afforded by mature vessels may create a permissive niche for fibroblast proliferation. Masson's trichrome staining confirmed accelerated collagen fiber deposition in the LPA-treated wounds on day 9 (Fig. 5F).

**Fig. 6:**
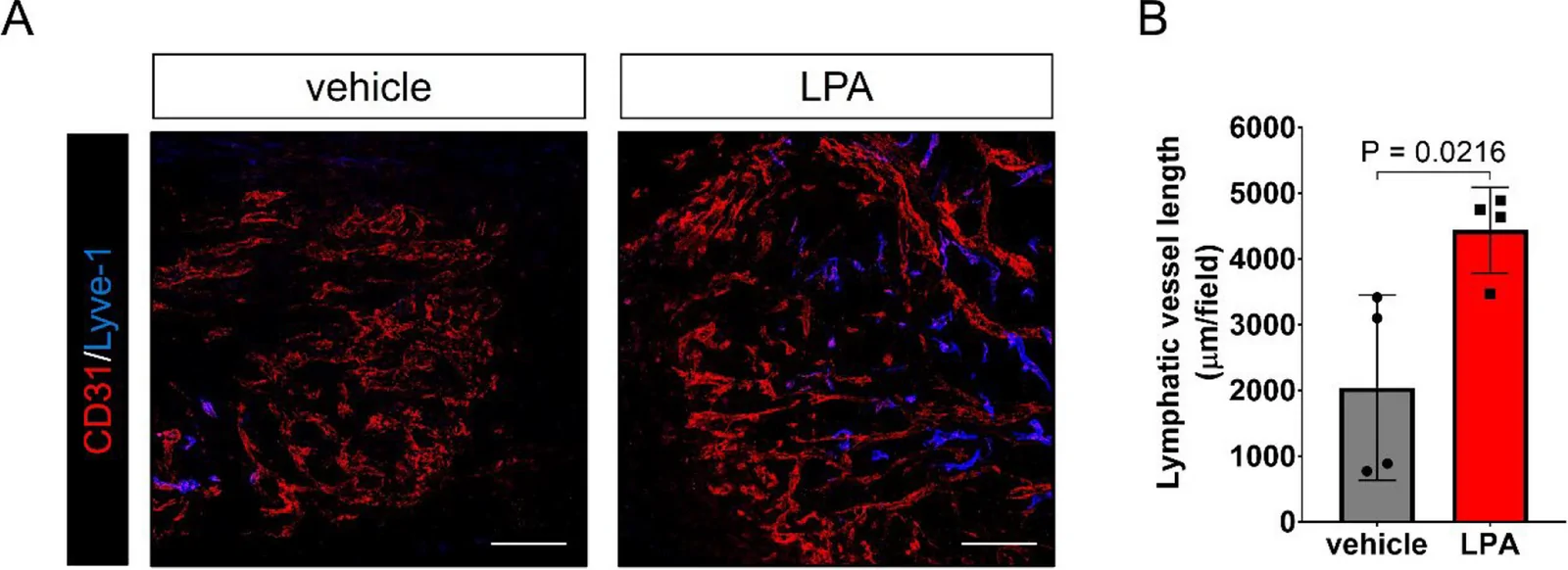
LPA promotes lymphangiogenesis at the wound site. **A** Representative confocal images of day 7 wound sections co-stained with CD31 (red) and LYVE-1 (blue), showing blood and lymphatic vessels in vehicle- and LPA-treated mice. Scale bar = 100 μm. **B** Quantification of lymphatic vessel length per field, measured from LYVE-1-positive structures in (**A**); n = 4; P = 0.0216. Data are presented as mean ± SD

We further examined whether LPA affected the lymphatic vasculature. Wound sections co-stained with CD31 and the lymphatic endothelial marker LYVE-1 revealed visibly increased LYVE-1⁺ lymphatic structures in LPA-treated wounds (Fig. 6A), and quantification confirmed a significant increase in lymphatic vessel length, relative to the vehicle (Fig. 6B). These findings demonstrate that the pro-regenerative activity of LPA extends beyond blood vessels to lymphangiogenesis.

### LPA receptor expression in wound-associated cell populations

**Fig. 7:**
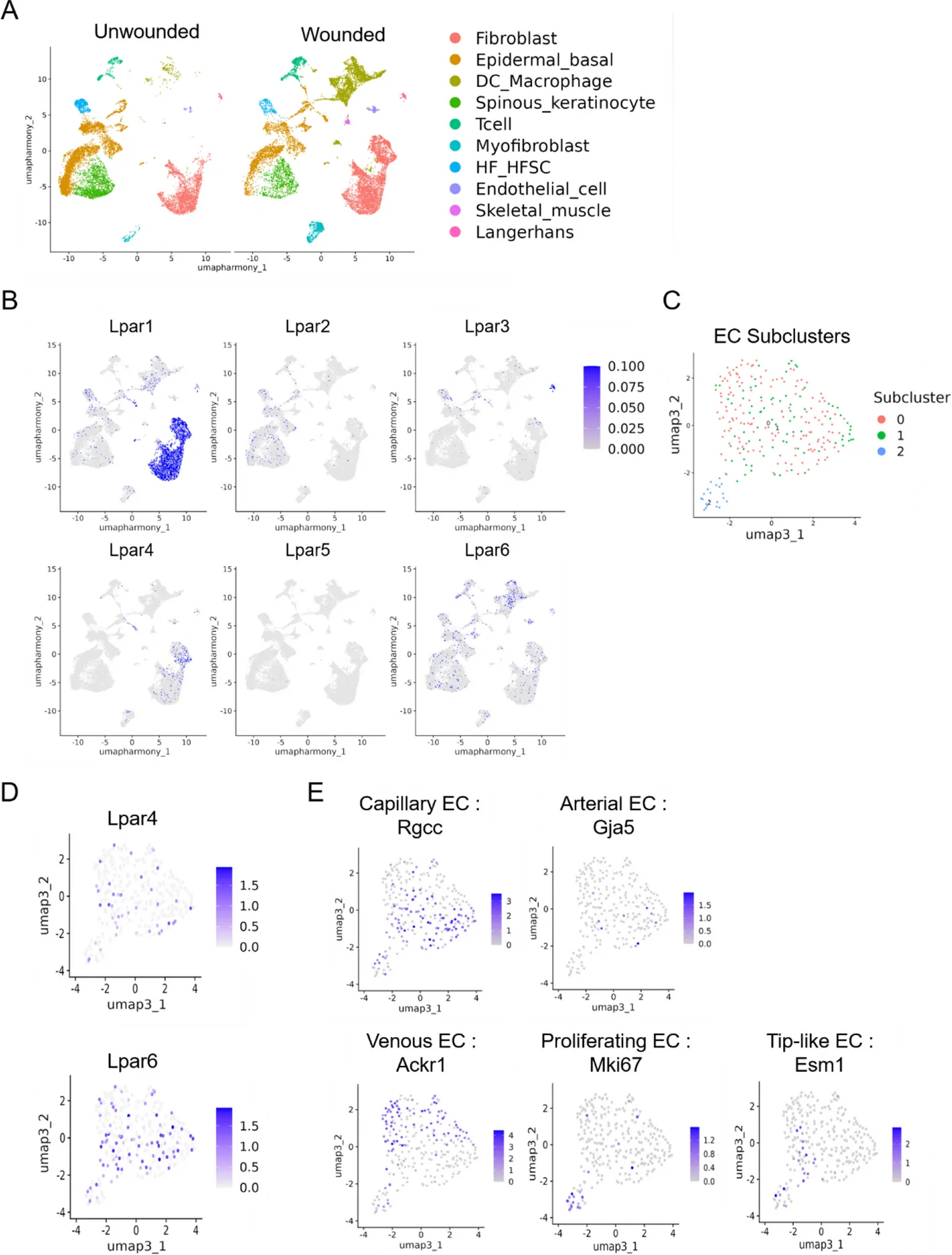
LPA receptors show distinct expression across wound-associated cell populations. **A** UMAP plot from re-analyzed single-cell RNA sequencing data of mouse whole skin showing 10 annotated cell populations under unwounded and wounded conditions. **B** Feature plots of LPAR1–6 transcript expression overlaid on UMAP. Color intensity indicates normalized expression level. LPAR1 is predominantly expressed in fibroblasts; LPAR4 and LPAR6 are enriched in endothelial cells. **C** UMAP plot of computationally isolated and re-clustered endothelial cells, resolving three EC subclusters. **D** Feature plots showing *Lpar4* and *Lpar6* expression across EC subclusters. **E** Feature plots showing the expression of established markers for capillary (*Rgcc*), arterial (*Gja5*), venous (*Ackr1*), proliferating (*Mki67*), and tip-like (*Esm1*) ECs

To map the cellular targets of LPA in the wound, we re-analyzed a published single-cell RNA sequencing dataset of whole mouse skin comprising unwounded and wounded conditions [21]. UMAP embedding resolved 10 major cell populations, including fibroblasts, epidermal basal cells, DCs/macrophages, spinous keratinocytes, T cells, myofibroblasts, hair follicle stem cells, ECs, skeletal muscle cells, and Langerhans cells (Fig. 7A). Feature plot analysis revealed that LPAR1 was most prominently expressed in fibroblasts, whereas LPAR4 and LPAR6 were enriched in ECs and LPAR2, LPAR3, and LPAR5 showed relatively low expression levels overall (Fig. 7B). To further resolve the distribution of *Lpar4* and *Lpar6* within the endothelial compartment, we computationally sub-clustered the EC population, which resolved three distinct subclusters (Fig. 7C). Annotation of these subclusters using established markers for capillary (*Rgcc*), arterial (*Gja5*), venous (*Ackr1*), proliferating (*Mki67*), and tip-like (*Esm1*) ECs showed that *Lpar4* and *Lpar6* were broadly expressed across all subtypes rather than being restricted to a specific subtype (Fig. 7D, E).

## Discussion

This study investigated the therapeutic potential of LPA in cutaneous wound repair using a murine full-thickness excisional wound model, with a particular focus on its effects on vascular and stromal remodeling. Wounding itself induced a transient increase in *Enpp2* (ATX) expression, and pharmacological inhibition of ATX modestly delayed wound closure during the earliest phase of healing, suggesting that endogenous LPA signaling contributes to the reparative response (Supplementary Fig. 1). This effect diminished at later time points, consistent with the transient nature of *Enpp2* induction, after which other growth factors, such as VEGF, are likely to become the predominant drivers of tissue regeneration.

Functional angiogenesis is central to cutaneous wound repair; however, neovasculature quality is as important as its quantity. Structurally immature and highly permeable vessels fail to support efficient tissue perfusion and may regress without contributing to sustained repair. VEGF, the most potent known driver of angiogenesis, robustly expands the endothelial compartment but typically generates leaky pericyte-poor vessels prone to such regression [22].

In the present study, LPA-induced vessels showed elevated pericyte coverage on day 7, followed by reduced permeability and suppressed tissue hypoxia on day 13. This temporal sequence was consistent with the progressive vascular maturation that preceded and accompanied the accelerated wound closure observed in this model. These features—enhanced pericyte recruitment, decreased extravasation, and improved tissue oxygenation—are canonical hallmarks of structurally and functionally mature vasculature. This finding represents a conceptual shift from traditional pro-angiogenic approaches: rather than simply expanding vessel number, LPA promotes the formation of vessels that are functionally competent to support tissue repair. The vascular maturation mechanism likely involves LPAR4 and/or LPAR6 in ECs, as suggested by the single-cell receptor mapping. Indeed, our previous work in tumor models established that LPAR4 signaling induces circumferential actin bundling and VE-cadherin-mediated linear adherens junction formation in ECs, thereby tightening intercellular junctions and reducing vascular permeability [12]. A similar LPAR4- and/or LPAR6-driven mechanism may contribute to vascular stabilization in the wound microenvironment, although direct evidence from receptor-selective or genetic perturbation experiments in this context requires further investigation. Apelin and angiopoietin-1 have been reported to promote vascular stabilization in wounds and tumors [23, 24]; however, LPA appears to be unique in simultaneously expanding vessel number and driving their functional maturation, an effect we previously observed in tumor vasculature and is now demonstrated in the physiological setting of wound repair.

The transcriptome-level data independently corroborated these findings. Cluster 4 genes upregulated by LPA on days 5 and 7 spanned vasculature development and ECM organization, respectively. This was mirrored by transient but significant upregulation of fibronectin, Col1a1, Col3a1, and Tgfb mRNA levels and by accelerated collagen deposition on Masson's trichrome staining. The temporal pattern, with peak expression on day 5, followed by normalization toward day 9, was consistent with a well-regulated, productive fibroblast activation wave, rather than a sustained fibrotic response. Notably, *Lpar1* expression itself remained stable through day 9 (Supplementary Fig. 3), indicating that the decline in matrix gene expression was unlikely to result from loss of receptor expression. Instead, this transient response may reflect reduced penetration of topically applied LPA following re-epithelialization or a physiological transition into the remodeling phase, which is characterized by upregulation of matrix-degrading enzymes and myofibroblast apoptosis. Masson's trichrome staining on day 9 revealed markedly thickened granulation tissue with abundant collagen fiber deposition in LPA-treated wounds, consistent with an active and accelerated tissue formation response. Nonetheless, the long-term assessment of scar quality will be an important goal in future studies. The proliferating perivascular ER-TR7-positive cells are consistent with fibroblasts responding to the improved tissue oxygenation afforded by mature vasculature, and single-cell data showing LPAR1 expression in fibroblasts raise the additional possibility of a direct LPA-LPAR1 axis that operates independently of vascular effects. Dissecting the relative contributions of these two pathways, vascular-mediated and direct receptor-mediated, will be an important goal for future studies.

The induction of lymphangiogenesis by LPA is a noteworthy additional finding. The lymphatic vasculature facilitates interstitial fluid drainage and resolution of edema during tissue repair [25], and agents that co-stimulate both vascular systems are uncommon. This dual activity may contribute to the qualitative improvement in tissue restoration observed following LPA treatment. A recent study reported that LPA signaling through LPAR4 and LPAR6 regulates lymphatic EC behavior, including aspects of lymphatic valve biology [26]. Whether analogous receptor-mediated pathways contribute to lymphangiogenesis in wounds remains unclear. Nevertheless, the dual promotion of blood and lymphatic vessel growth by LPA may contribute to more complete tissue restoration, as adequate lymphatic drainage is required for resolution of wound edema and immune cell egress. Taken together, these observations highlight that LPA exerts diverse effects on multiple cell types, including blood vascular ECs, lymphatic ECs, and fibroblasts. However, the precise mechanisms through which LPA acts on these cell populations in a wound context remain to be elucidated. In particular, the extent to which the effects on fibroblasts and lymphatic ECs are mediated directly through LPA receptors expressed on these cells, or indirectly through improved tissue oxygenation resulting from vascular maturation, remains unclear. Conditional knockout models for individual LPAR genes in blood vascular ECs, lymphatic ECs, fibroblasts, and immune cells are required to dissect these receptor-cell type axes and to definitively assign causality to each pathway.

Although the present findings highlight the therapeutic potential of LPA in wound repair, including its sustained efficacy even when treatment is initiated several days after injury (Supplementary Fig. 4), clinical translation will require careful consideration of the pleiotropic nature of LPA signaling. LPA promotes tumor cell proliferation and survival through the PI3K/Akt and MAPK pathways and fosters an immunosuppressive tumor microenvironment by suppressing CD8^+^ T cell function and M2 macrophage polarization, raising concerns about tumor progression in susceptible patients [27]. LPAR1 signaling has also been implicated in the initiation of neuropathic pain through demyelination of peripheral neurons, and in promoting neuroinflammation, raising concerns regarding potential neurotoxicity or chronic pain development with systemic administration [28]. Furthermore, LPA promotes platelet aggregation via LPAR1 and LPAR5 and amplifies inflammation within atherosclerotic plaques, which may increase the risk of pro-thrombotic events and adverse cardiovascular events [29].

This study has some limitations. Although LPA did not promote tumor growth in our previous mouse experiments, caution is warranted for use in patients with active malignancies, until dedicated safety data are available. Notably, the topical route of administration employed in the present study may inherently limit systemic exposure and thereby mitigate some off-target risks, representing a practical advantage over systemic delivery. Taken together, these considerations demonstrate that the path toward clinical application will require validation in large animal wound models and that the development of receptor subtype-selective agonists or organ-targeted delivery systems that harness the pro-healing effects of LPA while minimizing systemic off-target risks.

## Conclusions

This study demonstrates that topical LPA accelerates cutaneous wound healing through concurrent induction of functionally mature vasculature and fibroblast-driven ECM deposition, with additional promotion of lymphangiogenesis. These findings establish LPA as a mechanistically distinct therapeutic candidate that targets vascular maturation, rather than angiogenic expansion, indicating a novel class of agents in cutaneous wound care. Further translational studies are warranted to evaluate its therapeutic potential in chronic wound settings.

## Supplementary Information


Supplementary Material 1.

## Data Availability

The bulk RNA sequencing datasets generated in this study have been deposited in the NCBI Gene Expression Omnibus (GEO) under accession number [PRJDB42128] and are publicly available. The single-cell RNA sequencing data re-analyzed in this study were obtained from a previously published dataset (GEO accession: GSE166950). All other data supporting the findings are available from the corresponding author upon reasonable request.

## Abbreviations

αSMA: Alpha-smooth muscle actin
ATX: Autotaxin
BSA: Bovine serum albumin
ECM: Extracellular matrix
EGF: Epidermal growth factor
EC: Endothelial cell
FGF: Fibroblast growth factor
FITC: Fluorescein isothiocyanate
GEO: Gene Expression Omnibus
GO: Gene Ontology
H&E: Hematoxylin and eosin
LPA: Lysophosphatidic acid
LPAR: Lysophosphatidic acid receptor
LYVE-1: Lymphatic vessel endothelial hyaluronan receptor 1
PDGF: Platelet-derived growth factor
qRT-PCR: Quantitative reverse transcription polymerase chain reaction
RNA-seq: RNA sequencing
scRNA-seq: Single-cell RNA sequencing
tSNE: T-distributed stochastic neighbor embedding
UMAP: Uniform Manifold Approximation and Projection
VEGF: Vascular endothelial growth factor
