# Supplementary material for: Lysophosphatidic acid promotes cutaneous wound healing through vascular maturation and fibroblast activation in a murine model
Source: Inflamm Regen. 2026 Aug 28;46:33. doi: 10.1186/s41232-026-00441-5 (PMC13523234; doi:10.1186/s41232-026-00441-5)
Supplement: Supplementary file 1 [file 41232_2026_441_MOESM1_ESM.docx]

**Supplemental Figure.1**

**Supplementary Figure 1. Wounding induces endogenous *Enpp2* (ATX) expression, and ATX inhibition delays wound healing.**
(A) Feature plot of *Enpp2* transcript expression overlaid on the UMAP embedding of re-analyzed single-cell RNA sequencing data from mouse skin, showing expression in endothelial cells, fibroblasts, myofibroblasts, and epidermal basal cells. (B) Quantitative RT-PCR of *Enpp2* expression (normalized to *Gapdh*) in unwounded skin and wound tissue at 3 h, 6 h, and days 1, 3, 5, 7, and 9 after wounding; n = 3 per group. (C) Representative photographs of wounds from vehicle- and PF-8380 (30 mg/kg, oral, once daily)-treated mice on days 0, 1, 3, 5, 7, 9, 11, and 13. Scale bar = 1 cm. (D) Time-course of wound area normalized to the day 0 value (mean ± SD; n = 6 per group). (E) Statistical comparison of wound area between the vehicle and PF-8380 groups on days 1, 3, 5, and 7. Data are presented as mean ± SD; n = 6 per group.

**Supplemental Figure.2**

**Supplementary Figure 2. LPA promotes VE-cadherin–based endothelial junctional maturation.**
(A) Representative confocal images of day 7 wound sections co-immunostained for VE-cadherin (green) and CD31 (red) in vehicle- and LPA-treated mice, with higher-magnification views of the boxed regions. Scale bars = 200 μm (low magnification) and 50 μm (high magnification). (B) Normalized fluorescence intensity profiles of VE-cadherin and CD31 along the white line shown in (A) for vehicle- and LPA-treated wounds.

**Supplemental Figure.3**

**Supplementary Figure 3. LPAR1 expression remains stable during wound healing.**
Quantitative RT-PCR of *Lpar1* expression (normalized to *Gapdh*) in wound tissue on days 5, 7, and 9; n = 3 per group. ns, not significant.

**Supplemental Figure.4**

**Supplementary Figure 4. Delayed initiation of topical LPA treatment remains effective.**
(A) Experimental timeline: topical LPA (1.0 mg/mL) or vehicle was applied once daily beginning on day 3 after wounding. (B) Representative photographs of wounds from vehicle- and LPA-treated mice on days 0, 1, 3, 5, 7, 9, 11, and 13. Scale bar = 1 cm. (C) Time-course of wound area normalized to the day 0 value (mean ± SD; n = 6-7 per group). (D) Statistical comparison of wound area between the vehicle and LPA groups on days 5, 7, and 9; n = 6–7 per group. Data are presented as mean ± SD.
